# Dissecting Duodenal Hematoma: A Rare but Serious Complication of Esophagogastroduodenoscopy

**DOI:** 10.1097/PG9.0000000000000052

**Published:** 2021-03-08

**Authors:** Brad D. Constant, Jason P. Weinman, Marisa G. Stahl

**Affiliations:** From the * Department of Pediatrics, The Digestive Health Institute, University of Colorado School of Medicine/Children’s Hospital Colorado, Aurora, CO and the; † Department of Radiology, University of Colorado School of Medicine/Children’s Hospital Colorado, Aurora, CO.

**FIGURE 1.**
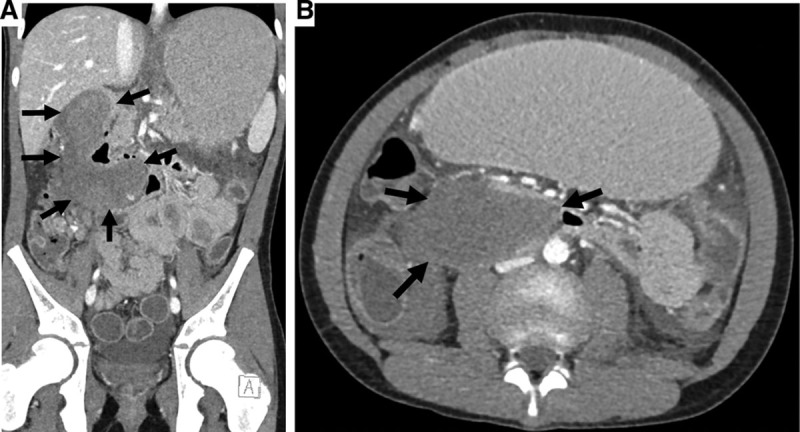
Coronal (A) and transverse (B) computed tomography of the abdomen and pelvis images showing an obstructive dissecting duodenal hematoma involving the second and third portions of the duodenum. Arrows delineating hematoma borders.

A 12-year-old boy presented to the emergency department with abdominal pain, abdominal distention, and nonbloody, nonbilious emesis 1 day after routine esophagogastroduodenoscopy (EGD) with biopsies to confirm suspected celiac disease, without known complication. Celiac disease was suspected based on gastrointestinal symptoms and elevated tissue transglutaminase IgA. Physical examination demonstrated epigastric tenderness, abdominal distention, and tachycardia. He was not malnourished (body mass index 17.4, Z-score −0.43). Labs were notable for a lipase concentration of 10,261 U/L (upper limit of normal = 195 U/L). Computed tomography of the abdomen and pelvis with oral and intravenous contrast revealed a dilated stomach and proximal duodenum, hemoperitoneum, and a dissecting intramural hematoma involving the second and third portions of the duodenum causing obstruction and pancreatitis (Fig. 1A: coronal and Fig. 1B: transverse; arrows delineating hematoma borders). The patient was transferred to a tertiary care center intensive care unit. Given the extent of the hematoma, evolving abdominal exam, hemodynamic instability, and concern for possible perforation, he underwent urgent exploratory laparoscopy and retroperitoneal duodenal hematoma evacuation without evidence of perforation within 24 hours of transfer. The hematoma improved on subsequent fluoroscopic imaging. Coagulopathy workup was negative. He recovered with bowel rest and decompression. He was discharged 2 weeks later after slow nutritional advancement to a liquid diet. Within a month of diagnosis, he was tolerating a full gluten-free diet with no residual symptoms.

Overall, post-EGD bleeding rates are only 0.11–0.3% in pediatrics (1,2). Duodenal hematomas make up a subset of these bleeds, occurring in only 0.05% of EGDs with biopsies (3). A pediatric case series of 14 patients with duodenal hematomas demonstrated that they are usually managed conservatively (14/14), although none were complicated by duodenal dissection. This case series identified coagulopathy and organ transplant recipient status as possible risk factors. Coagulopathy related to malnutrition and vitamin K deficiency is a risk factor for endoscopic bleeds; however, our patient’s internationalized normalized ratio was normal, presumably indicating this did not contribute to his presentation (4). While celiac disease has been hypothesized to be a risk factor for endoscopic complications, this has not been confirmed and represents an area in need of future research.

This case highlights a rare complication of routine EGD with biopsies, which, in this scenario, necessitated surgical intervention. Although rare, the possibility of duodenal dissection highlights the need to continually balance the risks and benefits of pursuing endoscopic confirmation of celiac disease, especially if ESPGHAN criteria for serologic diagnosis are met (5).
